# Radiation-Induced Brachial Plexopathy: Current Understanding, Diagnosis, and Treatment Options

**DOI:** 10.1016/j.jhsg.2025.100896

**Published:** 2025-12-05

**Authors:** Alex G. Lambi, Tomas Holy, Ryan E. Tomlinson, Mary F. Barbe

**Affiliations:** ∗ Department of Surgery Division of Plastic Surgery, University of New Mexico School of Medicine, Albuquerque, NM; † Department of Orthopedics and Rehabilitation, University of New Mexico School of Medicine, Albuquerque, NM; ‡ University of New Mexico Comprehensive Cancer Center, Albuquerque, NM; § Department of Surgery Plastic Surgery Section, New Mexico Veterans Affairs Health Science Center, Albuquerque, NM; ‖ Department of Orthopaedic Surgery, Thomas Jefferson University, Philadelphia, PA; ¶ Aging and Cardiovascular Discovery Center, Lewis Katz School of Medicine at Temple University, Philadelphia, PA

**Keywords:** Brachial plexus, Fibrosis, Plexopathy, Radiotherapy, RIBP

## Abstract

Radiation-induced brachial plexopathy (RIBP) is a gruesome complication of cancers treated with radiation therapy around the lung and chest wall, head and neck, and breast and axilla. It can occur in an early-onset (transient) or a late-onset (chronic) fashion. The diagnosis involves exclusion of a compressive neoplastic process, either new or recurrent, and relies largely on patient symptomatology without well-validated, objective scoring systems. Treatment options remain limited as no major advances have been made to prevent or halt disease progression. This article reviews the background incidence, pathophysiology, and diagnosis of RIBP. In addition to surgical treatment options, nonsurgical modalities, often the mainstay of symptom management, are discussed. Lastly, the current challenges in treating RIBP are highlighted with an emphasis on targeting the underlying culprit—radiation-induced fibrosis.

Radiation-induced brachial plexopathy (RIBP) is a morbid complication of cancers treated with radiotherapy (XRT).^1^^,^^2^ Initially described in breast cancer patients treated with adjuvant XRT, it can also be seen after XRT in head, neck, lung, and chest wall cancers.^3^, ^4^, ^5^ The RIBP presentations include early-onset (transient) and late-onset (chronic), with symptoms appearing months to years following XRT.^5^, ^6^, ^7^, ^8^ Diagnosing RIBP is challenging because of the difficulty in differentiating it from cancerous spread with involvement of the brachial plexus.^6^ Beyond diagnosis, severe RIBP remains a dreaded complication because of the limited treatment options and low likelihood of symptom improvement, especially as life expectancies have increased with improvements in cancer treatments.^9^ This review article will focus on the incidence, pathophysiology, clinical history, and diagnosis of RIBP and expands on an excellent review by Mosa et al.^10^ In addition, we review the surgical and nonsurgical therapies for RIBP, highlighting the critical need for new strategies to treat radiation-induced fibrosis.

## Incidence and Risk Factors

The primary risk factor associated with RIBP is the total radiation dose used during XRT. This occurs in a dose-dependent manner. The highest reported annual incidence of RIBP occurs in breast cancer patients, at approximately 2.9%, with the risk remaining largely constant throughout a patient’s lifetime. Yet the incidence has varied in the literature, with initial incidences reported as high as 15% and 66% in women having received 51 Gy and 60 Gy of total XRT dose, respectively.^5^ Improvement in XRT techniques has lowered total radiation doses to <55 Gy, likely contributing to the lower incidence of RIBP.^7^^,^^9^^,^^11^^,^^12^ More recent studies suggest an incidence closer to 1.0% to 1.2% in breast cancer patients, with 80% of cases being early-onset, resolving spontaneously within the year.^7^^,^^13^^,^^14^ Other RIBP incidences are estimated to be 7% in all head and neck cancers, specifically 0.3% in nasopharyngeal cancers.^11^^,^^15^, ^16^, ^17^, ^18^, ^19^

Radiation doses >50 Gy have been shown to cause damage to the neural tissue, though risk factors specific to radiotherapy technique, such as large total dose, short source-to-skin distance, and salvage radiotherapy in previously treated areas, have been identified.^13^^,^^20^, ^21^, ^22^, ^23^ In addition to the total radiation dose, adjuvant or prior neurotoxic chemotherapeutic agents (eg, cisplatin, vinca alkaloids, taxanes), concomitant surgery because of hematoma or chronic infection, and extended lymph node dissections have been identified as notable risk factors for RIBP.^21^^,^^24^ Potential patient-related factors include age (patients are generally either younger or elderly), obesity, diabetes, tobacco use, peripheral neuropathy secondary to other causes, and chronic diseases (eg, rheumatoid arthritis, systemic lupus erythematosus).^20^^,^^24^ Finally, underlying inflammatory and autoimmune damage to the plexus may predispose to a higher risk of XRT-related complications.^25^

## Pathophysiology and Natural History

External beam radiotherapy is the most common type of XRT administered for cancer treatment, with roughly 50% of all cancer patients receiving XRT in their treatment course.^26^ For the purposes of understanding the clinical manifestations of RIBP, a brief discussion of radiation-induced pathophysiology is included. For an extensive discussion of radiotherapy toxicity in all tissue types, see an in-depth review by De Ruysscher et al.^25^ Modern external radiotherapy beams can be targeted at the submillimeter level. Nonetheless, normal noncancerous tissue is often affected. Radiation-induced tissue damage causes both early and late effects in the brachial plexus. As a result, RIBP is typically categorized as either early-onset (transient) or late-onset (chronic) (Table 1).^1^^,^^2^^,^^7^^,^^13^, ^14^, ^15^^,^^20^^,^^27^, ^28^, ^29^, ^30^, ^31^, ^32^, ^33^, ^34^, ^35^, ^36^, ^37^, ^38^, ^39^, ^40^, ^41^, ^42^, ^43^, ^44^, ^45^, ^46^

**Table 1:** RIBP Categories and Associated Findings

RIBP Category	Time of Onset (Post-XRT)	Tissue Effects	Clinical Findings	Electrodiagnostic Findings	Resolution	Ref.
Early-onset (transient)	▪ 4–6 mo^13^^,^^15^^,^^27^	▪ Altered vascular permeability, inflammation, demyelination, and Schwann cell loss^28^ ▪ Edema-induced nerve compression^20^^,^^29^^,^^30^	▪ Axillary and neck pain ▪ Distal paresthesia ▪ Motor weakness ▪ Symptoms plateau after 3–6 months^20^^,^^27^	▪ Slowed conduction velocities^31^^,^^32^ ▪ Resolves with clinical course^13^^,^^20^	▪ 80% spontaneous resolution^7^^,^^13^^,^^14^	^7^^,^^13^, ^14^, ^15^^,^^20^^,^^27^, ^28^, ^29^, ^30^, ^31^, ^32^
Late-onset (chronic)	▪ 3–4 y^1^^,^^2^^,^^20^^,^^33^	▪ Chronic inflammation and fibrosis ▪ Tissue atrophy, microvascular damage, and demyelination^20^^,^^34^^,^^35^ ▪ Fibrosis-induced nerve constriction^32^^,^^36^, ^37^, ^38^, ^39^	▪ Paresthesia and sensory decline ▪ Weakness, progressive motor deficits, atrophy, and paralysis^2^^,^^20^^,^^37^^,^^40^^,^^41^^,^^43^^,^^44^ ▪ Hypo- or areflexia ▪ Lymphedema and fibroatrophic skin^41^	▪ Proximal conduction blocks ▪ Decreased motor and sensory actional potentials ▪ Myokymic changes^42^, ^43^, ^44^, ^45^, ^46^	▪ Irreversible^20^^,^^34^^,^^35^	^1^^,^^2^^,^^20^^,^^34^, ^35^, ^36^, ^37^, ^38^, ^39^, ^40^, ^41^, ^42^, ^43^, ^44^, ^45^, ^46^

### Early-onset RIBP

Early tissue damage following XRT includes cell death and local inflammation. The effects on tissues vary by tissue type, with examples including hair follicles and dermis accounting for alopecia and dermatitis, respectively.^47^ In peripheral nerves, acute changes include altered vascular permeability, demyelination, loss of Schwann cells, and transient electrophysiological changes.^28^ Postradiation edema occurs, resulting in extrinsic nerve compression and damage.^20^^,^^29^^,^^30^

Early-onset RIBP typically occurs within the first 4 to 6 months following XRT, although it can occur up to a year. Initial symptoms include distal paresthesias in conjunction with local neck and axillary pain. Motor deficits can also be present, chiefly weakness. Neuropathic symptoms often worsen initially, reach a plateau for 3 to 6 months, and then undergo a period of improvement. Approximately 80% of these cases spontaneously resolve.^7^^,^^13^^,^^15^^,^^20^^,^^27^^,^^29^^,^^30^

### Late-onset RIBP

Persistent, chronic inflammation following XRT leads to late effects, such as tissue atrophy, microvascular damage, demyelination, and radiation-induced fibrosis (RIF).^20^^,^^34^^,^^35^ These effects are typically irreversible. The RIF occurs in a complex process involving both noncellular and cellular factors. Signaling molecules such as transforming growth factor-beta 1, connective tissue growth factor, interleukin-6, macrophage inflammatory protein-1 alpha, and monocyte chemoattractant protein-1 induce a fibrogenic response. Transforming growth factor-beta 1 and connective tissue growth factor, in particular, play a key role in the attraction of fibroblasts and conversion to myofibroblasts, the integral cell type in fibrosis.^36^^,^^48^, ^49^, ^50^, ^51^, ^52^ A critical difficulty in the resolution (or treatment) of RIF is that once the local extracellular matrix becomes cross-linked, it becomes extremely resistant to the degradative breakdown needed for restorative repair.^53^^,^^54^ At the peripheral nerve level, RIF includes extraneural and intraneural fibrosis and neural hypoxia (from microvascular damage), ultimately leading to significant constriction, conduction blockage, and impairment of function.^34^^,^^36^, ^37^, ^38^, ^39^

Late-onset RIBP is the more common presentation of RIBP and becomes symptomatic an average of 40 months after XRT. However, presentation time is highly variable, from several years to over a decade after treatment.^1^^,^^2^^,^^20^^,^^33^ Symptoms of late-onset RIBP are more insidious. Patients typically present with paresthesia and pain in the affected extremity, followed by subsequent weakness and limb atrophy.^1^^,^^2^ Sensation may progressively decline to hypesthesia and even complete absence. Deep tendon reflexes may fade and become absent. Amyotrophy and motor deficits often ensue after sensory changes, with not uncommon progression to complete extremity paralysis.^20^^,^^36^^,^^38^, ^39^, ^40^, ^41^ When limb paralysis occurs, it is typically within several months to 5 years after the first signs of hand paralysis.^20^^,^^40^

## Patient Presentation and Diagnosis

Patient symptoms on presentation reflect the nature and degree of the radiation damage to the brachial plexus, including the chronicity and the extent of the plexus involved. The RIBP most frequently affects the entire brachial plexus (56% of the time), followed by variations of the superior plexus from C5–C8 (44%).^4^ Initial symptoms that prompt a patient to see a hand or peripheral nerve specialist may mimic carpal tunnel syndrome, though with pathophysiology initiating from the brachial plexus, symptoms will gradually broaden to include the forearm and arm.^20^^,^^40^ Furthermore, given the length of progression of late-onset RIBP and its persistent nature, “second hit” phenomena from other neurologic conditions or distal injuries may result in rapidly worsening symptoms and neurologic function because of the tenuous nature of brachial plexus function proximally.^20^ A comprehensive history and detailed physical examination are necessary to define the involved nerve distribution. Inspection of affected myotomes may reveal muscle fasciculations. A Hoffmann-Tinel percussion test in the axilla and/or supraclavicular region may exacerbate paresthesia and pain.^20^^,^^40^ Lymphedema and fibroatrophic skin changes accompany nerve symptoms in 20% of late-onset RIBP cases.^41^

Diagnosis of RIBP is a combination of clinical suspicion and subsequent exclusion of the differential diagnoses. The patterns of nerve involvement are often diagnostic and helpful in excluding other potential etiologies of brachial plexopathy in the setting of XRT following cancer treatment. Two other such causes include index tumor recurrence or invasion and radiation-induced neoplasm development.^22^ The different symptomatology and timelines of these processes often help distinguish clinically. Neoplastic brachial plexopathy is more often overtly symptomatic early in the clinical course and primarily affects the lower brachial plexus trunks, with features including Horner syndrome and intense pain. Conversely, RIBP tends to affect the entire plexus or superior aspect of the plexus (upper and middle trunks) and presents in a comparatively delayed fashion (as discussed previously), with accompanying symptoms such as painlessness and lymphedema.^1^^,^^2^^,^^6^^,^^55^^,^^56^

No single unifying scoring system exists that captures the pathophysiologic and physical findings and provides guidance for disease monitoring and treatments. The most cited scoring system is the late effects of normal tissues (LENT), subjective, objective, management, and analytic (SOMA).^57^^,^^58^ Specific LENT SOMA scales exist for many anatomic sites, including peripheral nerves, and are divided into four grades as described in Table 2.^59^ The analytic (“A” in SOMA) component does not delineate based on grade, further highlighting the lack of methods to measure disease progression.

**Table 2:** LENT SOMA Scoring

	Grade 1	Grade 2	Grade 3	Grade 4	Scoring	
**SUBJECTIVE**						
Pain	Occasional and minimal	Intermittent and tolerable	Persistent and tolerable	Refractory and excruciating		
Strength		Detectable weakness	Persistent weakness	Paralysis, transverse myelitis		
Sensory	Occasional paresthesia, hyperesthesia	Intermittent paresthesia	Persistent paresthesia	Paralysis		
Motor paresis	Occasional	<50% decrease from baseline capabilities	≥50% decrease from baseline capabilities	Paralysis		
**OBJECTIVE**						
Motor dysfunction	<20% loss	20% to 30% loss	30% to 50% loss	>50% loss		
Sensory dysfunction	Paresthesia	Vibration decrease	Decrease to pin prick	Complete anesthesia		
Reflex	Decreased deep tendon reflex	Absent deep tendon reflex				
**MANAGEMENT**						
Pain	Occasional non-narcotic	Regular non- narcotic	Regular narcotic	Surgical intervention		
Motor dysfunction			Physical or medical intervention	Surgical intervention		
Sensory dysfunction			Physical or medical intervention	Surgical intervention		
Sensory				Neurosurgical intervention		
					Total “SOM” score	
					LENT score (total score divided by 11)	
**ANALYTIC**						
MRI	Assessment of associated muscle atrophy, changes in nerve intensity				Yes/No	Date of study:
Nerve conduction studies	Assessment of speed, absence of conduction of electrical impulse				Yes/No	Date of study:

Adapted from “Peripheral Nerves” in *Int J Radiat Oncol Biol Phys***31**, 1049-1091.^59^ Scoring is performed by assigning each of the 11 SOM parameters a number 0–4. Score a 0 if there are no applicable toxicities. Score 1–4 based on the matching Grade description for each parameter. The LENT score is the total of these parameter scores divided by 11. The analytic responses are answered yes or no and are not factored into the LENT score.

## Imaging and Electrodiagnostic Studies

While clinical history and examination are the primary diagnostic methods in patients with RIBP, magnetic resonance imaging (MRI) and electrodiagnostic studies (EDX) are commonly performed to further characterize the extent of disease.^20^^,^^41^ In all organs, changes following XRT are not specific to any one imaging modality to date.^25^ RIBP-associated fibrosis typically appears as a thickened plexus on MRI, with distorted architecture, and a nonenhancing signal intensity similar to skeletal muscle on both T1- and T2-weighted images. The MRI is useful in excluding a neoplastic process, which often shows the presence of a mass and hyperintensity on T2-weighted images after gadolinium injection.^56^^,^^60^^,^^61^ In the event of potential neoplastic invasion, additional studies (eg, Positron emission tomography/CT) may be required based on specific oncologic criteria.^62^ Additional imaging tests are being developed to assess nerve fibrosis. One such example is ultrasound shear-wave elastography, which has shown early promise in identifying increased nerve stiffness in diseases such as RIBP.^63^, ^64^, ^65^, ^66^, ^67^, ^68^, ^69^, ^70^

EDX, including EMG and nerve conduction studies (NCS), are supportive in the overall diagnosis of RIBP, delineating the location and extent of plexus involvement and helping to rule out neoplastic plexopathy; 90% of patients with either RIBP or neoplastic plexopathy will have conduction abnormalities on EDX.^71^^,^^72^ Findings in early-onset RIBP show slowing of conduction velocities on NCS as underlying transient demyelination occurs; however, this typically resolves spontaneously in concert with the clinical course.^13^^,^^31^^,^^32^ As late-onset RIPB progresses, changes in the sensory and motor potentials can be seen. Proximal conduction blocks, because of focal demyelination and/or fibrotic compression, occur. Distal muscle groups (eg, thenar) may show motor potentials of diminishing amplitude. Detection of motor units may be present in the absence of gross function for years.^42^ A key difference between the two is the detection of myokymic changes (underlying spontaneous muscle discharges) and fine quivering (undulating movements) in RIBP. These changes present on EMG as recurrent single motor unit potential bursts firing at 5 to 150 Hz.^43^, ^44^, ^45^, ^46^ Myokymia is rarely seen (in 4%) of neoplastic plexopathies—compared to 60% of RIBP cases.^2^^,^^31^^,^^32^

## Nonsurgical Treatment

The current treatment goal of both early- and late-onset RIBP is symptom management. No large trials have been performed with preventative therapies given prior to or during XRT. Studies using pharmacotherapies in other XRT-related side effects, such as dermatitis, have been performed without significant clinical benefit.^73^ Historically, occupational and physical therapy have been advocated throughout the disease process with the aims of maintaining joint range of motion, preserving muscle function, and reducing lymphedema.^22^^,^^74^^,^^75^ Nevertheless, care should be taken to avoid stretching the plexus against surrounding fibrosis, excessive ranges of motion, and carrying heavy loads to minimize further neurologic insult.^20^^,^^76^

Pharmacotherapies used in RIBP treatment are targeted at patient symptoms, chiefly pain management. Given the chronic nature of the neuropathic pain, benzodiazepines, tricyclic antidepressants, and antiepileptics are often used to augment pain control.^20^^,^^41^ Antiepileptics, specifically membrane-stabilizing ones such as carbamazepine, may reduce nerve hyperexcitability.^1^^,^^20^ Although quinine has been proposed for treating muscle cramps, the FDA removed this as an approved indication given the risk of serious adverse effects.^77^

Currently, no treatments are available that have been shown to slow or prevent the progression of symptomatic plexopathy. Corticosteroids are frequently used in the treatment of patients with acute radiation toxicity (eg, dermatitis), but no large studies have shown a benefit in preventing long-term nerve damage.^25^^,^^76^ Anticoagulants have been suggested to provide benefit by limiting nerve ischemia, though with limited results.^78^ A promising target in the underlying pathophysiology of RIBP is fibrosis itself, a biologic process most consider irreversible once present in tissues. Several currently available anti-inflammatory and antifibrotic pharmacotherapies exist but have yet to be tested in and approved for radiation-induced nerve damage.^79^ Interestingly, a combination therapy of pentoxifylline and tocopherol (vitamin E) has shown promise in treating RIF elsewhere in the body. However, a recent clinical trial comparing this regimen to a placebo failed to show a benefit in RIBP.^80^

Beyond pharmacotherapies, hyperbaric oxygen therapy (HBOT) has been suggested in RIBP management. HBOT has resulted in improved outcomes in several small trials on patients with late radiation tissue injury of the head and neck. However, no clinical effect on neurologic tissue was seen.^81^ When specifically considering HBOT in RIBP, some studies report a reduction in pain, edema, and erythema—but not of the underlying tissue fibrosis.^75^^,^^81^^,^^82^ In contrast, other studies have shown no clinical benefit from HBOT in RIBP.^81^^,^^83^^,^^84^

## Surgical Treatment

Surgical treatment of RIBP extends the goal of symptom management to ideally include functional improvement. Limited studies on surgical options and their success have been performed, with largely inconclusive results.^9^ The rationale for specific surgical interventions can be understood by the intended target of the procedure: the fibrotic peri-plexus wound bed or the nerve function itself. Ultimately, both are frequently addressed simultaneously at the time of surgery.

Surgical procedures on the tissue surrounding the plexus are largely aimed at removing constricting fibrotic tissue and restoring a healthy, vascularized wound bed. The most commonly reported and used procedure involves neurolysis and concurrent vascularized free tissue transfer with an omental flap.^85^^,^^86^ By surrounding affected nerves with healthy tissue and eliminating dead space, this procedure aims to reduce continued fibrosis and result in improved symptoms.^86^^,^^87^ Local and regional muscle and adipofascial flaps have also been described in smaller patient groups to minimize the requirements of free tissue transfer.^88^^,^^89^

However, these procedures have proven to be of limited broad benefit given unpredictable results. A recent systematic review demonstrated that roughly half (53%) of patients note improvements in pain, while a smaller percentage show functional improvements in motor (19%) and sensory (16%) symptoms. The remaining patients demonstrate either no change or worsening of their symptoms (8%). The latter may be explained by the trauma of surgery and subsequent scarring. In a subset of patients, gradual motor and sensory deterioration was noted despite improvements in pain.^4^^,^^39^^,^^90^ Given that motor weakness is a late finding in the pathophysiology of RIBP and therefore may portend severe to permanent nerve injury, neurologic recovery in the presence of motor weakness is unlikely in patients undergoing neurolysis. Ultimately, the main indication to perform surgical neurolysis with or without vascularized tissue transfer in RIBP is refractory pain uncontrolled or unresponsive to less invasive therapies.

In addition to attacking the hostile fibrotic peri-plexus environment, other functional procedures that bypass this zone of injury have been described in RIBP. The main example of this in the literature is using distal nerve transfers to restore biceps function and elbow flexion,^42^^,^^91^ with one study demonstrating recovery to MRC grade 4/5.^42^ The premise of performing distal nerve transfers relies on several important factors. First, enough plexus-innervated function must be present such that donor nerve transfer does not compromise clinical function. Next, it assumes that proximal nerve damage, including conduction loss, microvascular damage, and demyelination, does not affect the ability of the distal nerve to transmit function. This concept is not well supported by the literature. Finally, it requires intact neuromuscular endplates. Preoperative electrodiagnostic studies should be obtained prior to consideration of motor nerve transfer, as decreased or absent motor unit potentials in the target muscles portend poor outcomes for a nerve transfer. If a nerve transfer is performed, postoperative physical therapy and motor rehabilitation are crucial in cortical remapping and clinical improvement.^88^ Other functionally reconstructive procedures, such as free function muscle transfer, have been proposed, but their use remains limited given the paucity of available proximal donor nerves in RIBP.^92^

## Current Challenges and Future Directions

Incredible improvements in treating peripheral nerve injuries have been made since RIBP was initially described nearly 60 years ago. Concurrently, advances in screening and therapeutics have drastically improved survival after cancer, with an estimated 18 million cancer survivors currently living in the United States.^93^ XRT is the cornerstone of many cancer treatments, with half of all cancer patients requiring it during their treatment course.^26^ Progressive fibrosis (RIF) is a key feature of the permanent adverse effects of radiation. A critical difficulty in treating RIF is the resistance of fibrotic extracellular matrix to degradative breakdown. Unlike other fibrotic conditions (eg, idiopathic pulmonary fibrosis), where an early window for treatment prior to disease progression may not exist, XRT in cancer therapy is almost always planned. This fact reveals the greatest need in RIBP management—the development of therapies that could be administered during planned XRT to lessen or prevent RIF. Studying this at the basic science level remains limited as we lack a small animal model to reproduce RIF that results in neuromuscular functional deficits. There is a huge range in the literature regarding the irradiation dose needed to induce RIF in vivo (5–90 Gy).^34^^,^^48^^,^^94^ Therefore, establishing a reliable model is necessary before adjunct therapies in RIPB can be trialed. Examples of potential therapies include currently approved drugs in fibrosis and inflammation^79^ and the addition of adipose-derived stem cells from autologous fat transfer.^50^

## Conclusion

Radiation-induced brachial plexopathy remains a dreaded complication of radiation therapy, a component of roughly half of all cancer treatments. RIBP occurs in an early-onset, transient fashion or progresses to a late-onset, chronic process with clinical findings from intermittent paresthesia to complex sensory and motor loss. Once other causes of symptoms, chiefly a neoplastic process, have been excluded, treatment options remain limited. Despite nearly 60 years of progress in the surgical treatment of other peripheral nerve injuries and disorders, no major advances in surgical or nonsurgical treatments have been made to prevent or halt the progression of this devastating process. As we look toward future advances in this field, close attention needs to be paid to therapies targeting the underlying culprit—RIF. A key advantage of this strategy is that radiotherapy is a planned intervention.

Finally, we conclude with a popular maxim of Benjamin Franklin—“An ounce of prevention is worth a pound of cure”.^95^ Written in 1735 in support of city-wide fire prevention strategies, this tenet can be applied to RIBP—radiation is the fire, and developing new preventative therapies will stave off the scorched ruins of crippling pain and lost upper extremity function.

## Conflicts of Interest

No benefits in any form have been received or will be received related directly to this article.
